# Effects of limited water supply on metabolite composition in tomato fruits (*Solanum lycopersicum* L.) in two soils with different nutrient conditions

**DOI:** 10.3389/fpls.2022.983725

**Published:** 2022-09-08

**Authors:** Yangmin X. Kim, Su Young Son, Seulbi Lee, Yejin Lee, Jwakyung Sung, Choong Hwan Lee

**Affiliations:** ^1^National Institute of Agricultural Sciences, Rural Development Administration, Wanju, South Korea; ^2^Department of Bioscience and Biotechnology, Konkuk University, Seoul, South Korea; ^3^Department of Crop Science, College of Agriculture, Life and Environment Sciences, Chungbuk National University, Cheongju, South Korea; ^4^Research Institute for Bioactive-Metabolome Network, Konkuk University, Seoul, South Korea

**Keywords:** lycopene, metabolite profiling, mineral nutrient, tomato fruit quality, water

## Abstract

Effect of water supply to metabolites in tomato fruit was compared in two soils with different nutrient conditions, i.e., either limited or excess. Two types of soil nutrient condition, type A: nutrient-limited and type B: nutrient-excess, were prepared as follows; type A is a low nutrient-containing soil without a replenishment of starved nitrogen and phosphorous, type B is a high nutrient-containing soil exceeding the recommended fertilization. Soil water was adjusted either at −30 kPa (sufficient) or −80 kPa (limited). For harvested tomato fruits, we examined primary and secondary metabolites using non-targeted mass spectrometry based metabolomics. The fruit production and leaf SPAD were greatly dependent on soil nutrient levels, by contrast, the level of lycopene remained unchanged by different levels of water and nutrient supply. The perturbation of metabolites by water supply was clear in the nutrient-excess soil. In particular, limited water supply strongly decreased primary metabolites including sugars and amino acids. We demonstrated that water stress differently shifted primary metabolites of tomato fruits in two soils with different nutrient conditions *via* non-targeted mass spectrometry-based metabolomics. In conclusion, we suggest that the limited water supply in soils with surplus nutrient is not a recommendable way for tomato ‘cv. Super Dotaerang’ production if fruit nutritional quality such as sugars and amino acids is in the consideration, although there was no disadvantage in fruit yield.

## Introduction

Water stress is a major problem where water is scarce (Hasan et al., 2021) and proper irrigation is essential in the greenhouse. Limited soil moisture reduces plant growth due to limited water uptake, which leads to the decrease in leaf elongation and stomatal conductance that results in the reduction in photosynthesis (Bista et al., 2018). Limited soil moisture also affects plant growth by reducing soil nutrient supply (Végh, 1991). Nutrient uptake during the water stress may decrease by reducing nutrient supply through mineralization, by hindering nutrient transport in the soil, and by affecting the kinetics of nutrient uptake by roots (Bista et al., 2018). The nutrient uptake by roots occurs *via* mass flow along water using the apoplastic pathway and *via* membrane-bound transporters in the transcellular pathway (Bassirirad, 2000; Kim et al., 2018). Therefore, the stress response by limited soil moisture can interact with soil nutrient conditions as nutrients transport is related to the water transport (Roy et al., 2020).

Tomato fruit quality has been demonstrated to be modified by nutrient and water supply using metabolomics approach (Kwon et al., 2019; Gil et al., 2020; Kim et al., 2020). Gil et al. (2020) reported that tomato fruit quality was shifted by the amount of N supply, and lowering the N supply resulted in low amino acid levels, and it could be by the decrease in amino acid biosynthesis due to the N shortage (Causin, 1996). The Mg^2+^ surplus in the soil worsened fruit quality including sugars, organic acids and amino acids, and the metabolites in the leaves and roots were simultaneously compared, and they suggested that metabolites were allocated to the root (sink) and it resulted in unfavorable fruit quality (Kwon et al., 2019). Tomato fruits increased lycopene by reducing N supply after fruit setting in combination of sufficient water supply condition, when tomato was grown in the Mg^2+^ excess soil (Kim et al., 2020). On the other hand, deficit irrigation has been used as a strategy to enhance the crop quality and it increased the tomato fruit soluble solid, hexoses, citric acid, and potassium concentration (Mitchell et al., 1991). Nutrient excess such as Na^+^, Mg^2+^, and Ca^2+^ occurs in the greenhouse environment due to extensive application of fertilizers where rainfall does not reach to the soil surface. The nutrient excess causes harmful impact on plants by osmotic stress, in which reactive oxygen species is generated and it results in the oxidative stress to plant cells (Du et al., 2015). The nutrient excess in the soil hinders root water uptake by negative water potential in the soil and by changes in root anatomy and hydraulic properties (Adams et al., 2019).

In this study, we handled two types of soil nutrient condition, type A: nutrient-limited condition and type B: nutrient-excess condition. Considering the interaction between nutrient and water transport, we have expected the different tomato quality by deficit irrigation in the farm lands with different nutrient status. In that context, we tested whether imposing a moderate water stress to tomato compared to the sufficient water supply (control) would differently shift the primary and secondary metabolites in tomato fruits in two different soil nutrient conditions (type A and B). The purpose of this paper was to investigate how the limited irrigation in the soils containing different levels of nutrients affects the fruit nutrition of tomato growing in the glasshouse. In this study, we selected two extreme soil nutrient cases, nutrient excess and limited. Primary and secondary metabolites of tomato fruits were investigated using a non-targeted metabolomics. The metabolomics is a comprehensive approach to evaluate different metabolomes under a specific set of conditions (Son et al., 2016), and we have applied it for various crops in response to varying environmental factors including light and mineral nutrient supply (Kwon et al., 2019; Gil et al., 2020; Kim et al., 2020, 2021; Suh et al., 2020).

## Materials and methods

### Plant materials and nutrient and water supply

Two-month-old tomato seedlings (*Solanum lycopersicum* L., ‘Super Dotaerang’; Koregon Co., Ltd., Anseong, Korea) were transplanted into plastic pots (4.5 kg of soil) in March, 2019 and were grown for 18 weeks in the glasshouse in the National Institute of Agricultural Sciences, Rural Development Administration, Korea. The daily temperatures in the glasshouse was between 15–35°C and the heating and ventilating systems prevented extreme temperature conditions. There was no regulation in relative humidity of the glasshouse and the natural light penetrating the glasshouse was used for plant growth.

Two soils with different nutrient conditions were employed (Table 1) in order to set up the nutrient-limited soil condition and nutrient-excess soil condition during the plant growth. To produce the nutrient-limited soil condition during the plant growth, we did not supply nitrogen and phosphorous fertilizer to the low nutrient soil, while potassium chloride as a potassium fertilizer was supplied in accordance with the fertilizer recommendations for tomato cultivation in greenhouse soil (NIAS, 2017; Supplementary Table S1). To produce nutrient-excess soil condition during the plant growth, potassium fertilizer was applied to the high nutrient soil in accordance with the recommendations for tomato cultivation in greenhouse soil with high electrical conductivity, EC (NIAS, 2017). Three split applications of fertilizer were conducted by topdressing (Supplementary Table S1).

**Table 1:** Chemical properties of soils before transplanting tomato plants.

Soil	pH (1:5)	EC (1:5; dS m^−1^)	NO_3_-N (mg kg^−1^)	OM (g kg^−1^)	Av. P_2_O_5_ (mg kg^−1^)	Ex. Cations (cmol_c_ kg^−1^)		
						K	Ca	Mg
Low nutrient soil	6.3	0.8	75	15	172	0.09	2.9	0.6
High nutrient soil	5.9	8.6	871	32	791	1.69	13.3	5.0
Recommended^*^	6.0–6.5	<2.0	70–200	20–30	400–500	0.7–0.8	5.0–6.0	1.5–2.0

Mean values of three replicates.

* Recommended is the proper value for tomato cultivation in Korean plastic house.

Variations in water supply was from the 12th week from the transplanting that was after fruit setting; water was supplied either at −30 kPa for the sufficient supply or −80 kPa of soil water potential for the limited supply by reading tensiometers (Soilmoisture Equipment Corp., Santa Babara, United States). The gypsum block in tensiometer sensing the water potential was at the depth of 0.07–0.13 m below the soil surface. The water potential that hinders the plant growth is −100 kPa and permanent wilting point is −1,500 kPa. There were 4 soil × water supply conditions. Ripened fruits from 16 plants were harvested (four plants for each soil × water supply condition). Two to seven ripened tomato fruits from each plant were harvested from 28 June to 24 July, 2019.

### Measurement of leaf SPAD values

On the final day of harvesting, the SPAD values of the tomato leaves near the harvested fruits were measured using a chlorophyll meter (SPAD-502Plus, Konica Minolta Sensing, Osaka, Japan). SPAD values were measured as an indicator of the plant performance rather than an accurate value of chlorophyll content (Kim et al., 2020).

### Sample preparation and extraction

Sample preparation and extraction steps were adopted for our previous research (Kim et al., 2021). Cultivated tomato fruits under varied nutrient and water conditions were washed with distilled water and wiped before being stored at a deep freezer (−80°C). Frozen fruits samples were freeze-dried for 3 days at −98°C in lyophilizer (Operon, Gimpo, Korea) and grinded to powder using a mortar and pestle. Powdered samples (100 mg) were extracted with 80% methanol. To extract samples, all samples were homogenized with Retsch MM400 Mixer Mill (Retsch GmbH Co., Haan, Germany) and sonicated for 10 min. After then, extracted samples were centrifuged for 10 min at 12,000 rpm and 4°C (Gyrozen 1730R, Gyrozen Inc., Daejeon, Korea). The supernatants were filtered by polytetrafluoro-ethylene (Chromdisc, Daegu, Korea) and then filtered samples were completely dried using a speed vacuum concentrator for overnight. The final concentration of each analyzed samples was 20,000 ppm (20 mg/ml).

### GC-TOF-MS and UHPLC-LTQ-Orbitrap-MS analysis

For GC-TOF-MS analysis, the samples adjusted for concentration (100 μl) were dried again using a speed vacuum. The dried samples were derivatized with the following protocol. First step, 50 μl of methoxyamine hydrochloride (20 mg/ml in pyridine) was added to the dried samples and placed on the thermomixer for 90 min at 30°C. Then, 50 μl of N-methyl-N-trimethylsilyl trifluoroacetamide (MSTFA) was incubated for 30 min at 30°C. One microliter of each sample was injected at split mode (ratio 1: 20). The GC-TOF-MS instrument, analytical protocol, and parameter setting for analysis were adopted for our previous research (Kim et al., 2021).

For secondary metabolites, the re-dissolved samples were analyzed using UHPLC-LTQ-Orbitrap-MS equipped with Vanquish binary pump H system (Thermo Fisher Scientific, Waltham, MA, United States) coupled with an auto-sampler and column compartment. The analytical methods and operation parameters were followed by our previous study (Kim et al., 2021). All samples were run in a randomized manner to reduce bias and systematic errors.

### Lycopene analysis

The lycopene analysis including extraction method, analytical method and parameters, and operation setting was followed by our previous study (Kim et al., 2021; Mun et al., 2021). For lycopene extraction, powdered samples (200 mg) was extracted with 3 ml of solvent mixture (Chloroform: DCM = 2:1 (v/v)) coupled with orbital shaker (Biofree, Seoul, Korea) for 20 min and then 1 M sodium chloride solution (1 ml) added. The extracted samples were centrifuged at 5,000 rpm for 10 min at 4°C and the supernatants were completely concentrated using nitrogen gas. The concentrated samples were re-dissolved with methanol and tert-Butyl methyl ether solvent mixture (3:2, v/v). The level of lycopene was analyzed by liquid chromatography diode array detection system. Lycopene was identified with retention time and absorbance, which were compared to commercial standard compound of lycopene analyzed under identical states.

### Data processing and multivariate statistical analysis

MS data processing and multivariate statistical analysis were proceeded as described in our previous research (Kim et al., 2021; Mun et al., 2021). The raw data derived from GC-TOF-MS and UHPLC-LTQ-Orbitrap-MS were converted to netCDF (*.cdf) by chromaTOF (version 4.44, LECO Corp., St. Joseph, MI, United States) and Xcalibur (Version 2.00, ThermoFisher Scientific, Waltham, MA, United States) software, respectively. Then, the *.cdf files were performed with the Metalign software (RIKILT-Institute of Food Safety, Wageningen, The Netherlands) to determine peak mass value (m/z), retention time (min), baseline correction, and peak area normalization. After alignment, multivariate analysis was performed using final version of alignment data by SIMCA-P+ software (version 12.0, Umetrics, Umea, Sweden). Multivariate analysis such as principal component analysis (PCA) and partial least squares-discriminant analysis (PLS-DA) was proceeded to determine significantly distinguished metabolites between tomato cultivated under varied nutrient and water conditions. The significance of PLS-DA model was determined by analysis of variance testing of cross-validated predictive residuals (CV-ANOVA) came from SIMCA-P+ software. Distinguished metabolites were determined according to variable importance in the projection (VIP) value from PLS-DA model. Metabolites derived from GC-TOF-MS and UHPLC-LTQ-Orbitrap-MS analysis were tentatively identified by comparing mass spectrum, mass fragment pattern, retention time with available database including Wiley 9, the Human Metabolome Database (HMDB)^1^, the National Institute of Standards and Technology (NIST) database (Version 2.0, 2001, FairCom, Gaitherburg, MD, United States), in house database, previous research datasets, and standard compounds analyzed with identical condition. Significant differences were evaluated by Student’s *t*-test using PASW Statistics 18 software (SPSS Inc., Chicago, IL, United States).

### Statistical analysis

Significant differences in the production and lycopene content of ripened fruit and leaf SPAD values were evaluated by a one-way analysis of variance (ANOVA) followed by Tukey’s test (*p* < 0.05).

## Results

### Tomato production, lycopene contents of tomato fruits, and leaf SPAD

The fruit yield, lycopene content in fruits and leaf SPAD values were measured to compare the effects of water supply in the nutrient-limited and nutrient-excess soils (Table 2). The fruit production was significantly reduced in the nutrient-limited soil compared to nutrient-excess, when water supply was sufficient. The level of lycopene, a major functional metabolite of tomato fruit, on a dry-weight basis did not differ from both different conditions of water and nutrient of soils. The leaf SPAD values were significantly lower in tomato plants grown in the nutrient-limited soil than in nutrient-excess soil.

**Table 2:** Tomato fruit production per plant, lycopene contents of tomato fruits on a dry-weight basis, and leaf SPAD cultivated under varied nutrient and water conditions.

Soil nutrient	Water	Abbreviated name	Ripened tomato fruit production (g plant^−1^)	Ripened tomato fruit lycopene content (ppm)	Tomato leaf SPAD
Limited	Sufficient	NL-WS	189 ± 32b	889 ± 185a	22.3 ± 4.7b
	Limited	NL-WL	215 ± 14b	943 ± 453a	24.4 ± 4.5b
Excess	Sufficient	NE-WS	364 ± 100a	1,069 ± 212a	48.9 ± 10.4a
	Limited	NE-WL	237 ± 35a,b	960 ± 666a	49.9 ± 6.0a

Mean values ± SD of three biological replicates.

Different alphabetical letters in a column indicate significant differences, as determined by ANOVA followed by Tukey’s tests (*p* < 0.05). NL, nutrient limited; WS, water sufficient; WL, water limited; NE, nutrient excess.

### Non-targeted metabolite profiling of tomato fruits cultivated under different water supply in two soil nutrient conditions

**Figure 1:**
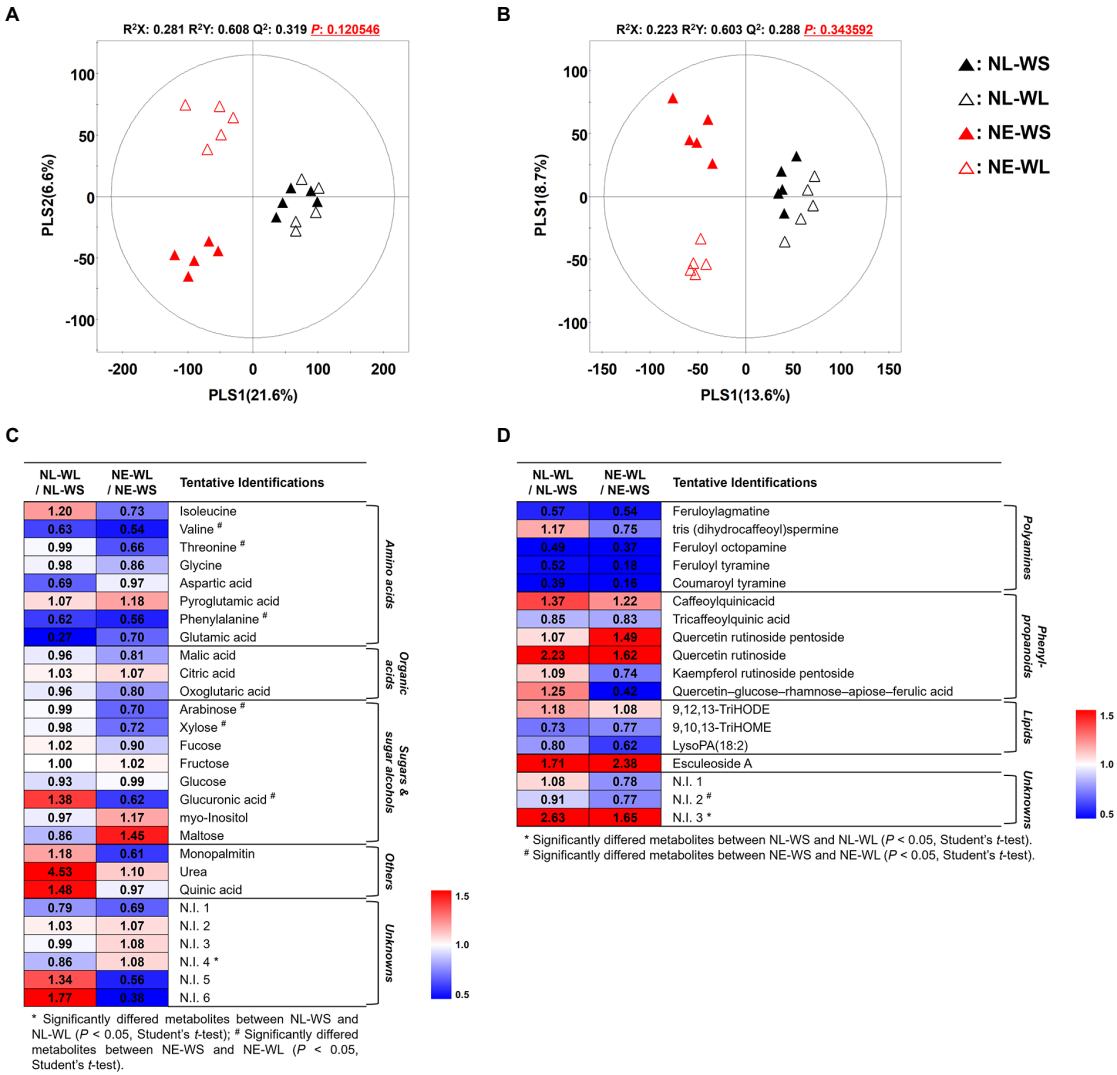
PLS-DA score plots from GC-TOF-MS **(A)** and UHPLC-LTQ-Orbitrap-MS **(B)** analysis of tomato cultivated under varied nutrient and water conditions. Symbol, NL-WS (▲); NL-WL (△); NE-WS (▲); NE-WL (△). Heatmap of the relative changes of metabolites by the water limitation for each soil nutrient condition for the distinguished metabolites derived from GC-TOF-MS **(C)** and UHPLC-LTQ-Orbitrap-MS **(D)** datasets (VIP > 0.7). The colored squares (blue to red) indicate fold changes normalized by water sufficient condition for each soil nutrient condition.

To determine global metabolites including primary and secondary metabolites in tomato fruits cultivated under different water supply in two different soil nutrient conditions, metabolites profiling approach was performed by GC-TOF-MS and UHPLC-LTQ-Orbitrap-MS/MS. Subsequently, multivariate analysis including unsupervised PCA, as well as the supervised PLS-DA were performed to determine the distinguished metabolites. The PLS-DA score plots based on GC-TOF-MS and UHPLC-LTQ-Orbitrap-MS/MS showed distinct pattern between nutrient-limited and nutrient-excess soil by PLS1 (21.6% and 13.6%, respectively) and water supply (limited and sufficient) was divided by PLS2 (6.6% and 8.7%, respectively) (Figures 1A,B). The statistical model value of PLS-DA was determined by R^2^X (0.281 and 0.223), R^2^Y (0.608 and 0.603) and Q2 (0.319 and 0.288), which indicated the model prediction accuracy, fitness, and validation. Moreover, the PCA score plot showed a similar pattern to the PLS-DA (Supplementary Figures S1A,B). Distinguished metabolites among the four groups were identified and selected based on variable importance in projection (VIP) value (>0.7) from the PLS-DA model and Student’s *t*-test was applied to determine statistical significance (*p* < 0.05).

A total of 47 discriminant metabolites were identified, of which 28 and 19 metabolites were determined by GC-TOF-MS and UHPLC-LTQ-Orbitrap-MS/MS, respectively (Supplementary Tables S2, S3). A total of 28 primary metabolites were determined by GC-TOF-MS analysis and categorized into the following metabolite classes including 8 amino acids *viz.*, isoleucine, valine, threonine, glycine, aspartic acid, pyroglutamic acid, phenylalanine, and glutamic acid, 3 organic acids *viz.*, malic acid, citric acid, and oxoglutaric acid, 8 sugars and sugar alcohols *viz.*, arabinose, xylose, fucose, fructose, glucose, glucuronic acid, myo-Inositol, and maltose, 3 others *viz.*, monopalmitin, urea, and quinic acid. Moreover, 19 secondary metabolite were tentatively identified by UHPLC-LTQ-Orbitrap-MS/MS and divided into sub-classes such as 5 polyamines *viz.*, feruloylagmatine, tris(dihydrocaffeoyl)spermine, feruloyl octopamine, feruloyl tyramine, and coumaroyl tyramine, 6 phenylpropanoids *viz.*, caffeoylquinicacid, tricaffeoylquinic acid, quercetin rutinoside pentoside, quercetin rutinoside, kaempferol rutinoside pentoside, and quercetin–glucose–rhamnose–apiose–ferulic acid, 1 alkaloids (esculeoside A), and 3 lipids *viz.*, 9,12,13-TriHODE, 9,10,13-TriHOME, and lysoPA (18:2).

The relative changes in metabolite levels by limited water supply were compared to sufficient water supply conditions in each nutrient condition and they were presented as a heatmap (Figures 1C,D). According to the heatmap analysis, the limited water condition in nutrient-excess soil (NE-WL) led to reduction in amino acids except for pyroglutamic acid, organic acids such as malic acid and oxoglutaric acid, and sugars and sugar alcohols except for fructose, myo-Inositol, and maltose. Notably, amino acids (valine, threonine, and phenylalaine) and sugars and sugar alcohols (arabinose, xylose, and glucuronic acid) were significantly decreased compared to the sufficient water condition in nutrient-excess soil (NE-WS). In case of secondary metabolites from nutrient-excess soil condition, all secondary metabolites did not show statistical differences between NE-WL and NE-WS. Polyamines and lipids except for 9, 12, 13-TriHODE decreased in NE-WL compared to NE-WS. Conversely, phenylpropanoids (except for tricaffeoylquinic acid, kaempferol rutinoside pentoside and quercetin–glucose–rhamnose–apiose–ferulic acid) and esculeoside A increased in NE-WL. On the other hand, identified and selected metabolite from the limited water condition in nutrient-limited soil (NL-WL) and the sufficient water in nutrient-limited soil (NL-WS) showed no significant difference, but there were metabolites that showed a different pattern from nutrient-excess soil groups. Isoleucine, sugars and sugar alcohols (fucose, glucuronic acid, myo-Inositol, and maltose), others (monopalmitin, urea, and quinic acid), tris (dihydrocaffeoyl)spermine, and phenylpropanoids (kaempferol rutinoside pentoside and quercetin–glucose–rhamnose–apiose–ferulic acid) showed opposite characteristic pattern from the nutrient-excess soil groups.

### Metabolic pathway anaylsis in two soil nutrient conditions

**Figure 2:**
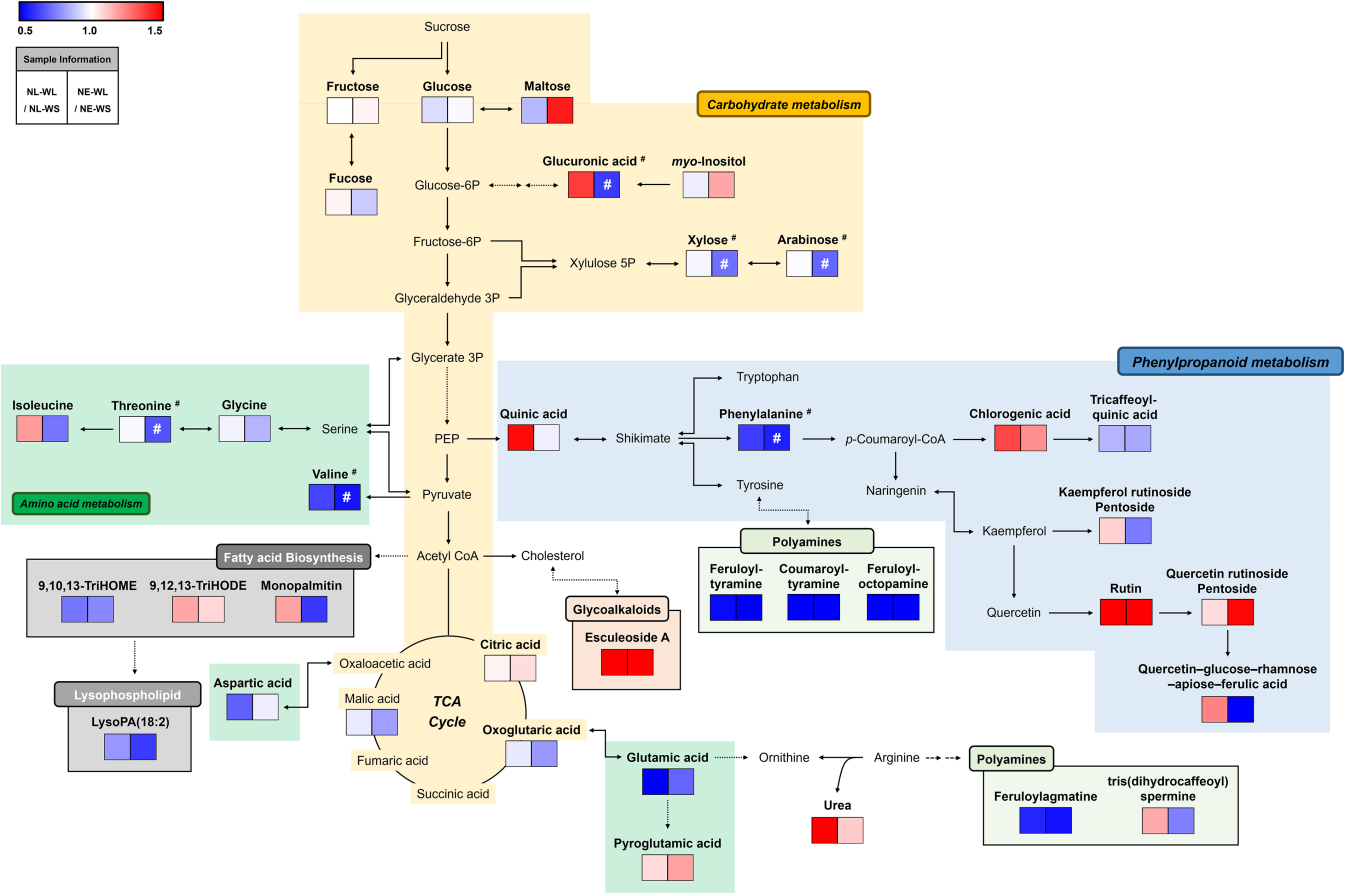
Schematic diagram of the metabolic pathway analysis and the relative changes in metabolites by water limitation in two different soils. The metabolic pathway was modified from the KEGG pathway database (https://www.genome.jp/kegg/pathway.html; URL accessed on 17 February 2022). The colored squares (blue to red) indicate fold changed normalized by water sufficient condition for each soil nutrient condition. ^#^Significantly differed metabolites between NE-WS and NE-WL (*p* < 0.05, Student’s *t*-test).

We performed a metabolic pathway analysis to understand the overall pattern of distinguished metabolites and the correlation between metabolites and water/nutrient conditions (Figure 2). The relative changes in metabolite levels by limited water (WL) compared to sufficient water (WS) condition for each nutrient condition (NL or NE) were presented in the pathway map. According to pathway map, amino acids-related metabolism was down-regulated in NL-WL and NE-WL compared to control conditions (NL-WS and NE-WS) except for pyroglutamic acid and isoleucine. Most of polyamines, organic acids, and sugar and sugar alcohols were also down-regulated in NL-WL and NE-WL compared to control conditions (NL-WS and NE-WS), respectively. Especially, myo-inositol and maltose were down-regulated only in NE-WL compared to control (NE-WS). In contrast, most of phenylpropanoids-related metabolism and glycoalkaloids (esculeoside A) were highly accumulated in NL-WL and NE-WL compared to control conditions (NL-WS and NE-WS) except for tricaffeoylquinic acid, kaempferol rutinoside pentoside, and quercetin–glucose–rhamnose–apiose–ferulic acid. Intriguingly, glucuronic acid, monopalmitin, urea, quinic acid, and tris(dihydrocaffeoyl)spermine were accumulated in NL-WL compared to control (NL-WS) but they were little accumulated or reduced in NE-WL compared to control (NE-WS).

## Discussion

In this study, tomato fruit yield was not significantly affected by the water conditions, limited or sufficient. Our result is similar that the tomato fruit yield grown in Mg^2+^ surplus soil was not changed by water limitation by regulating the soil water potential at the same level using cv. Super Dotaerang (Kim et al., 2020). In the current study, tomato fruit yield was decreased in the nutrient-limited soil compared to the nutrient-excess soil. The leaf SPAD value did not vary by the regulation in water supply in the two soil nutrient conditions, but leaf SPAD values in nutrient-limited soil were lower than those in nutrient-excess soil. The low SPAD value in the nutrient-limited soil is comparable to the result that the tomato leaf SPAD grown in Mg^2+^ surplus soil was significantly lowered by lowering N supply (Kim et al., 2020). The shoot sizes in nutrient-limited soil were smaller than those in nutrient-excess soil (Supplementary Figure S2), and it suggests that the overall growth of tomato plants was significantly different in two different soils. The content of lycopene, a major functional metabolite of tomato fruit did not vary not only by the regulation in water supply but also by soil nutrient conditions, contrasting to the big differences in the tomato shoot traits (i.e., leaf SPAD and shoot size) in two soil nutrient conditions. In the literature, the tomato lycopene content is known to be affected by irrigation and fertilization (Mitchell et al., 1991; Stefanelli et al., 2010; Wang and Xing, 2017; Kim et al., 2020; see below for further discussion on lycopene). Overall, we can underline that the water limitation in the current study was not a level to affect the fruit yield and leaf SPAD.

In our study, primary and secondary metabolites did not change substantially by water stress in the nutrient-limited soil, although there was a tendency that the water stress, low water potential (−80 kPa), accumulated phenylpropanoids and reduced polyamines, but there was no significant changes in metabolites. Indeed, water limitation is likely to induce a stress response, since phenylpropanoid is known to be upregulated as a response of plants against stress (Kim et al., 2019). In the nutrient-excess soil of our study, the water stress significantly decreased the primary metabolites including amino acids and sugars and there was a tendency of increase in phenylpropanoids and decrease in polyamines. The decrease in sugars in response to water stress in the nutrient-excess soil was in line with tomato fruits (cv. Super Dotaerang) grown in Mg^2+^ excess soil (Kim et al., 2020). As Kim et al. (2020) suggested, limited water was not recommended to improve tomato fruit (cv. Super Dotaerang) quality in nutrient-excess soil. In the current study, as a focus on improving the quality of tomato fruits (cv. Super Dotaerang) in terms of sugars, amino acids, and polyamines, limited water was not appropriate especially in nutrient-excess soil.

In this study, we tested only one cultivar, Super Dotaerang that is widely cultivated in the plastic house of South Korea and we left a room for other tomato cultivars that may show different responses. The cultivar dependent differences in chemical composition of tomato fruits from four landraces have been demonstrated in response to biostimulant (Rouphael et al., 2021). Effects of abiotic stress on tomato primary and secondary metabolites are known to be variable depending on cultivars (Quinet et al., 2019). For example, water stress increased sugars and organic acids of tomato in many cultivars, however, sugars and organic acids were not affected or decreased in some cultivars (Quinet et al., 2019). The effect of water stress for 141 accessions of diverse small fruit tomato was investigated, and 55 accessions out them exhibited increased metabolite contents in the fresh weight basis and maintained the yield (Albert et al., 2016b). Albert et al. (2016a) investigated 119 recombinant inbred lines derived from a cross between a cherry tomato and a large fruit tomato in response to water deficit (decrease by 60% compared to the control), and they observed more reduction in plant height and fruit production and greater increase in soluble solid contents in larger fruits. Therefore, they suggested that large fruit tomatoes could improve tomato flavor with small yield loss by a slight water deficit. In response to moderate salt stress, Meza et al. (2020) compared three tomato varieties and their fruits had different amino acid content. Flores et al. (2016) investigated the metabolites in tomato fruits in response to saline stress and they compared between a dwarf tomato cultivar and a commercial cultivar. In their study, two cultivars similarly increased carotenoids by saline stress, however, there were slight differences, i.e., the dwarf cultivar had lower capacity to accumulate primary metabolites including sugars and organic acids than the commercial cultivar; the dwarf tomato did not accumulate phenolic compounds and vitamin C differently from the commercial one. Therefore, it is deserved to endeavor to further explore the combined effects of nutrient and water supply condition on different tomato varieties. At least, current knowledge suggests that exploring different tomato varieties and cultivars in order to employ abiotic stresses as a cultivation technology is promising. To improve fruit quality with enhanced nutrients and functional metabolites, many studies have applied minimal irrigation to horticultural plants (Stefanelli et al., 2010). Deficit irrigation has been shown to improve the tomato fruit quality by increasing concentrations of hexoses, citric acid, and lycopene (Mitchell et al., 1991; Stefanelli et al., 2010; Wang and Xing, 2017). Beside of tomato, it was demonstrated that fruit quality increased under low irrigation conditions also in the cucumber (Zhang et al., 2011). In the future, we need to precisely define the cultivars and soil nutrient condition, when minimal irrigation would improve the crop quality, for practical application.

In terms of the interaction between nutrient and water, the limited water condition might have caused greater decrease in amino acids in tomato fruits in the nutrient-excess soil than in the nutrient-limited soil in our study. By a meta-analysis, water stress was demonstrated to decrease the concentration of nitrogen in plant tissue (He and Dijkstra, 2014). Decreases in nutrient concentration of plant tissues by drought was partly related to the decreases in the concentration of the root nutrient-uptake proteins (Bista et al., 2018). Nutrient availability to plant root is likely to be decreased by the shortage of available soil water, and nutrient uptake kinetics by the root could have been changed. We postulate that the nutrient uptake was substantially reduced by low soil moisture in the nutrient-excess soil. The cation contents such as Ca and Mg in the shoot of tomato plants grown in the nutrient-excess soil was also lower by limited water than that by sufficient water in the current study (data not shown). The combined effect of nutrient and water supply on tomato fruit quality was tested by Wang and Xing (2017). They applied three NPK fertilizer levels × three irrigation levels in a greenhouse and the highest nutrient × lowest water supply resulted in highest lycopene and organic acid content. Kim et al. (2020) investigated the effect of lowering either N or K supply in the combination of limiting water on quality of tomato fruit cultivated in Mg^2+^ excess soil. They showed the tomato fruit lycopene content was significantly lower in lowest N supply in the limiting water condition than in the sufficient water, while in other combinations the effect of limiting water supply was not significant.

## Conclusion

In summary, we examined the effects of moderate water stress on the quality of tomato fruits in two soils with different fertility, i.e., nutrient-limited and nutrient-excess. Non-targeted mass spectrometry-based metabolomics successfully demonstrated that water stress differently shifted metabolites of tomato fruits grown in two soils with different nutrient conditions. The limited water condition led to the significant reduction in the concentration of sugars and amino acids in only nutrient-excess soil. Therefore, we carefully suggest that the limited water supply is not recommended as a practical use to improve tomato (cv. Super Dotaerang) fruit quality including sugars and amino acids in soils with excess nutrients.

## Data availability statement

The original contributions presented in the study are included in the article/Supplementary material, further inquiries can be directed to the corresponding author.

## Author contributions

SL and JS: conceptualization. SL, YL, SS, and JS: validation. SL, YK, and SS: investigation. SL, YL, and JS: resources. YK and SS: writing—original draft preparation. YK, SS, YL, and JS: writing—review and editing. CL: supervision. SL, YL, and YK: project administration. SL and YL: funding acquisition. All authors contributed to the article and approved the submitted version.

## Funding

This research was funded by the “Cooperative Research Program for Agriculture Science & Technology Development (project no. PJ012523 and PJ014977)” and by 2022 RDA Fellowship Program of Rural Development Administration, Republic of Korea.

## Conflict of interest

The authors declare that the research was conducted in the absence of any commercial or financial relationships that could be construed as a potential conflict of interest.

## Publisher’s note

All claims expressed in this article are solely those of the authors and do not necessarily represent those of their affiliated organizations, or those of the publisher, the editors and the reviewers. Any product that may be evaluated in this article, or claim that may be made by its manufacturer, is not guaranteed or endorsed by the publisher.

## Footnotes

^1^http://www.hmdb.ca/ accessed on 17 of February 2022
