# Supplementary material for: Effects of limited water supply on metabolite composition in tomato fruits (Solanum lycopersicum L.) in two soils with different nutrient conditions
Source: Front Plant Sci. 2022 Sep 8;13:983725. doi: 10.3389/fpls.2022.983725 (PMC9492987; doi:10.3389/fpls.2022.983725)
Supplement: Supplementary file 1 [file Data_Sheet_1.docx]

Table S1. Fertilizer applications to tomato plants for two soils with different nutrient conditions

| Fertilizer  application | Time | Fertilization to low nutrient soil (g/kg soil) | Fertilization to high nutrient soil (g/kg soil) |
| --- | --- | --- | --- |
|  |  | K | K |
| 1^st^ supply | 1.5 months after transplant | 0.14 | 0.05 |
| 2^nd^ supply | 2 months after transplant | 0.14 | 0.05 |
| 3^rd^ supply | 3.5 months after transplant | 0.14 | 0.05 |

Table S2. Distinguished primary metabolites identified by GC-TOF-MS in tomato fruits cultivated under varied nutrient and water conditions

| **No.** | **Ret(min)^a^** | **VIP1** | **VIP2** | **Unique  mass(*m/z*)** | **Tentative Identifications^b^** | **Mass fragment pattern(*m/z*)** | **ID^c^** |
| --- | --- | --- | --- | --- | --- | --- | --- |
| ***Amino acids*** | | | | | | | |
| 1 | 6.10 | 1.91 | 1.38 | 86 | Isoleucine | 86, 75, 73, 69, 87, 146, 56, 61 | STD/MS |
| 2 | 6.49 | 1.90 | 1.65 | 144 | Valine ^#^ | 73, 144, 147, 77, 100, 218, 145, | STD/MS |
| 3 | 7.28 | 1.98 | 1.58 | 57 | Threonine ^#^ | 73, 117, 130, 147, 131, 132, 219 | STD/MS |
| 4 | 7.39 | 1.98 | 1.41 | 174 | Glycine | 73, 174, 86, 147, 100, 133, 248 | STD/MS |
| 5 | 9.29 | 1.95 | 1.40 | 232 | Aspartic acid | 73, 232, 100, 147, 117, 218 | STD/MS |
| 6 | 9.34 | 1.95 | 1.56 | 79 | Pyroglutamic acid | 73, 156, 75, 157, 147, 258, 230 | STD/MS |
| 7 | 10.16 | 1.99 | 1.63 | 192 | Phenylalanine ^#^ | 73, 218, 192, 100, 147, 219, 193 | STD/MS |
| 8 | 10.06 | 1.98 | 1.47 | 246 | Glutamic acid | 73, 246, 128, 147, 156, 100 | STD/MS |
| ***Organic acids*** | | | | | | | |
| 9 | 9.02 | 1.27 | 1.08 | 147 | Malic acid | 73, 147, 133, 55, 101, 233, 148 | STD/MS |
| 10 | 11.61 | 0.97 | 0.93 | 73 | Citric acid | 73, 147, 273, 149, 148, 274 | STD/MS |
| 11 | 11.84 | 1.79 | 1.59 | 173 | Oxoglutaric acid | 73, 55, 173, 157, 147, 129, 316 | STD/MS |
| ***Sugars & sugar alcohols*** | | | | | | | |
| 12 | 10.47 | 1.12 | 1.62 | 103 | Arabinose ^#^ | 73, 103, 147, 217, 117, 133, 307 | STD/MS |
| 13 | 10.42 | 1.14 | 1.63 | 103 | Xylose ^#^ | 73, 103, 217, 147, 133, 117 | STD/MS |
| 14 | 10.96 | 0.11 | 0.91 | 117 | Fucose | 73, 117, 147, 129, 89, 131, 215 | STD/MS |
| 15 | 12.02 | 1.30 | 0.93 | 344 | Fructose | 73, 103, 147, 217, 133, 307, 364 | STD/MS |
| 16 | 12.28 | 0.95 | 0.70 | 80 | Glucose | 73, 205, 160, 147, 103, 117, 319 | STD/MS |
| 17 | 12.53 | 1.57 | 1.59 | 333 | Glucuronic acid ^#^ | 73, 160, 147, 333, 143, 133, 189 | STD/MS |
| 18 | 13.44 | 0.65 | 0.98 | 191 | myo-Inositol | 73, 133, 103, 129, 204, 217, 319 | STD/MS |
| 19 | 17.03 | 1.28 | 1.76 | 191 | Maltose | 73, 147, 129, 103, 361, 217, 204 | STD/MS |
| ***Others*** | | | | | | | |
| 20 | 16.04 | 0.60 | 1.21 | 57 | Monopalmitin | 73, 147, 57, 129, 103, 117, 71 | STD/MS |
| 21 | 6.83 | 0.79 | 0.60 | 189 | Urea | 147, 189, 73, 171, 66, 79, 55, 99 | STD/MS |
| 22 | 11.92 | 1.40 | 1.05 | 345 | Quinic acid | 73, 147, 345, 255, 133, 191, 148 | STD/MS |
| ***Unknowns*** | | | | | | | |
| 23 | 9.54 | 1.59 | 1.24 | 120 | N.I. 1 | 120, 73, 146, 75, 130, 91, 103 | ‒^d^ |
| 24 | 12.70 | 0.92 | 0.66 | 169 | N.I. 2 | 73, 169, 231, 147, 59, 243, 148, 319 | ‒ |
| 25 | 12.84 | 1.17 | 0.85 | 158 | N.I. 3 | 73, 147, 205, 103, 129, 319, 117 | ‒ |
| 26 | 12.97 | 0.27 | 0.90 | 132 | N.I. 4 * | 73, 117, 75, 147, 129, 132, 103, 131 | ‒ |
| 27 | 13.76 | 1.59 | 1.31 | 172 | N.I. 5 | 73, 147, 103, 205, 157, 129, 319 | ‒ |
| 28 | 15.61 | 0.14 | 1.45 | 435 | N.I. 6 | 73, 147, 103, 75, 133, 435, 345, 89 | ‒ |

^a^ Retention time; ^b^ metabolites selected based on VIP (> 0.7) values obtained from PLS-DA model; ^c^ identification, STD/MS, comparison of mass spectrum with HMDB, NIST, wiley 9, and in house library and comparison with standard compounds analyzed under the same condition of GC-TOF-MS; ^d^ not detected. * Significantly differed metabolites between NL-WS and NL-WL (*p* < 0.05, Student’s *t*-test); # Significantly differed metabolites between NE-WS and NE-WL (*p* < 0.05, Student’s *t*-test).

Table S3. Distinguished secondary metabolites identified by UHPLC-LTQ-Orbitrap-MS in tomato fruits cultivated under varied nutrient and water conditions

| **No.** | **RT(min)^a^** | **VIP1** | **VIP2** | **[M‒H]^‒^** | **[M+H]^+^** | **Tentative identification^b^** | **Elemental  composition** | **Error (ppm)** | **MS fragment pattern (*m/z*)** | **ID^c^** |
| --- | --- | --- | --- | --- | --- | --- | --- | --- | --- | --- |
| ***Polyamines*** | | | | | | | | | | |
| 1 | 3.99 | 1.57 | 1.33 | 305.1656 | 307.1758 | Feruloylagmatine | C15H21N4O3 | -1.651 | (+)307> 290, 247, 177 | [REF, LIB] |
| 2 | 4.51 | 1.65 | 1.25 | 693.3618 | 695.3629 | Tris(dihydrocaffeoyl)spermine | C37H49N4O9 | -2.426 | 693> 571, 529> 407, 365 | [REF, LIB] |
| 3 | 4.86 | 1.11 | 1.32 | 328.1243 | 330.1329 | Feruloyl octopamine | (+)C18H20NO5 | -4.784 | 328> 310> 295, 252, 161, 135 | [REF, LIB] |
| 4 | 5.71 | 1.20 | 1.65 | 312.1331 | 314.1379 | Feruloyl tyramine | (+)C18H18NO4 | -1.318 | (+)312> 177, 144, 116 | [REF, LIB] |
| 5 | 5.51 | 1.37 | 1.70 | 282.1219 | 284.1272 | Coumaroyl tyramine | (+)C17H18NO3 | -4.646 | (+)284> 147, 121> 118 | [REF, LIB] |
| ***Phenylpropropanoids*** | | | | | | | | | | |
| 6 | 3.66 | 1.56 | 1.17 | 353.0921 | 355.1013 | Caffeoylquinicacid | C16H17O9 | -1.998 | 353> 191 | [REF, LIB] |
| 7 | 5.35 | 1.57 | 1.18 | 677.3005 | 679.2957 | Tricaffeoylquinic acid | C34H29O15 | -2.811 | 677> 515, 497 | [REF, LIB] |
| 8 | 4.43 | 0.99 | 1.07 | 741.1984 | 743.2014 | Quercetin rutinoside pentoside | C32H37O20 | 3.607 | 741>723, 609, 300> 271, 255 | [REF, LIB] |
| 9 | 4.60 | 0.41 | 0.87 | 609.1555 | 611.1593 | Quercetin rutinoside | C27H29O16 | 4.321 | 609> 301, 300> 271, 255, 179, 151 | [REF, LIB] |
| 10 | 4.60 | 0.84 | 0.81 | 725.2039 | 727.2072 | Kaempferol rutinoside pentoside | C32H37O19 | 3.914 | 725> 593, 575> 357, 327, 285 | [REF, LIB] |
| 11 | 5.06 | 0.70 | 1.05 | 917.2486 | 919.2496 | Quercetin–glucose–rhamnose–apiose–ferulic acid | C42H45O23 | -1.985 | 917>741, 723> 609, 591, 300 | [REF, LIB] |
| ***Alkaloids*** | | | | | | | | | | |
| 12 | 4.77 | 1.38 | 1.05 | 1268.6061 | 1270.6033 | Esculeoside A | C58H94NO29 | -4.350 | 1269> 1037, 1108, 975, 932> 914, 752, 590 | [REF, LIB] |
| ***Lipids*** | | | | | | | | | | |
| 13 | 6.15 | 1.22 | 1.01 | 327.2202 | 351.2137^d^ | 9,12,13-TriHODE | C18H31O5 | -0.637 | 327> 309, 291, 283, 229, 171 | [REF, LIB] |
| 14 | 6.44 | 0.34 | 0.85 | 329.2323 | 353.2287^d^ | 9,10,13-TriHOME | C18H34O5 | -3.090 | 329> 311, 293, 201, 171, 139 | [REF, LIB] |
| 16 | 8.99 | 0.01 | 0.87 | 433.2391 | 435.2491 | LysoPA(18:2) | C21H38O7P | 6.386 | 433>153 | [REF, LIB] |
| ***Unknowns*** | | | | | | | | | | |
| 17 | 3.97 | 0.89 | 0.93 | 445.177 | 469.1667^d^ | N.I. 1 | ‒^e^ | ‒ | ‒ | ‒ |
| 18 | 4.25 | 1.19 | 1.40 | 415.173 | 439.1559^d^ | N.I. 2 # | ‒ | ‒ | ‒ | ‒ |
| 19 | 6.15 | 2.19 | 1.61 | 271.0647 | 273.0753 | N.I. 3 * | ‒ | ‒ | ‒ | ‒ |

^a^ Retention time; ^b^ metabolites selected based on VIP (> 0.7) values obtained from PLS-DA model; c identification, REF, comparison of previous research datasets and in house library; ^d^ not detected. * Significantly differed metabolites between NL-WS and NL-WL (*p* < 0.05, Student’s t-test); # Significantly differed metabolites between NE-WS and NE-WL (*p* < 0.05, Student’s t-test).


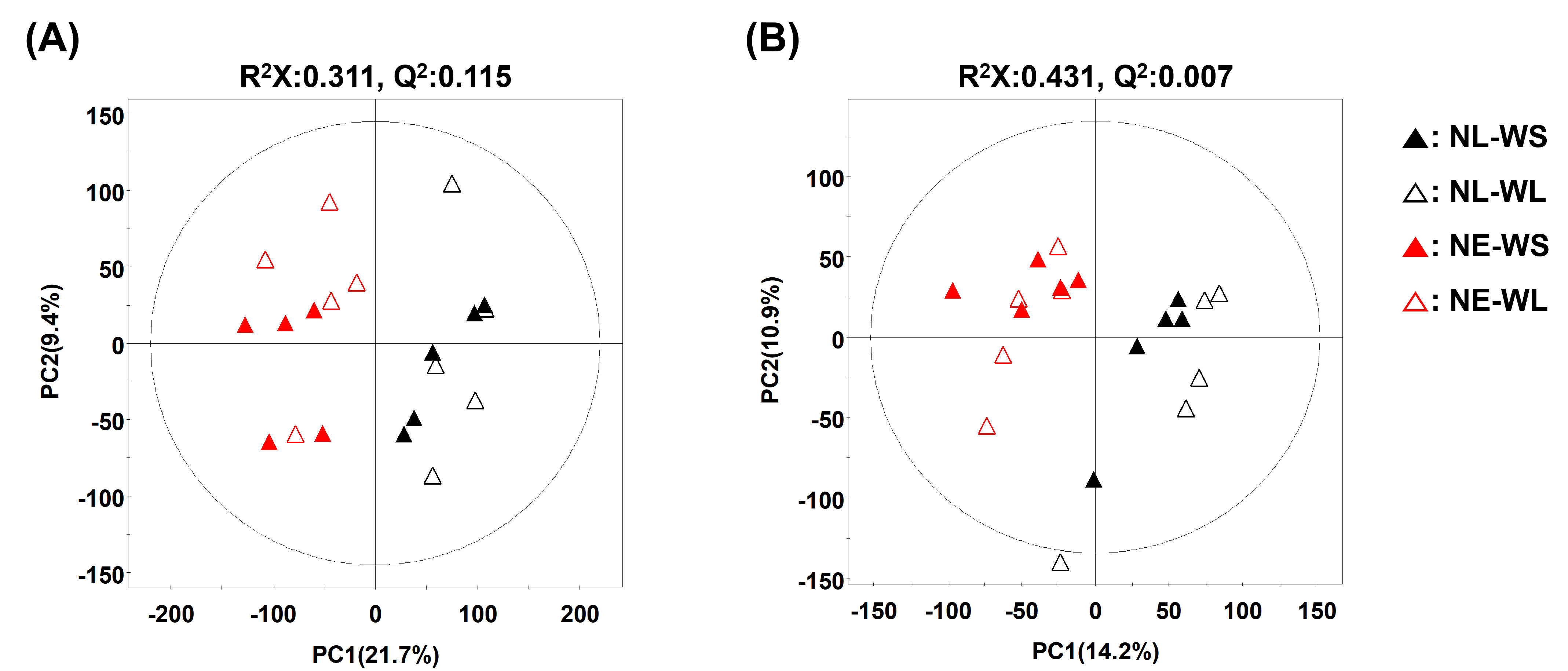


**Figure S1.** PCA score plots from GC-TOF-MS (A) and UHPLC-LTQ-Orbitrap-MS (B) analysis of tomato cultivated under varied nutrient and water conditions. Symbol, NL-WS (▲); NL-WL (△); NE-WS (▲); NE-WL (△).

| 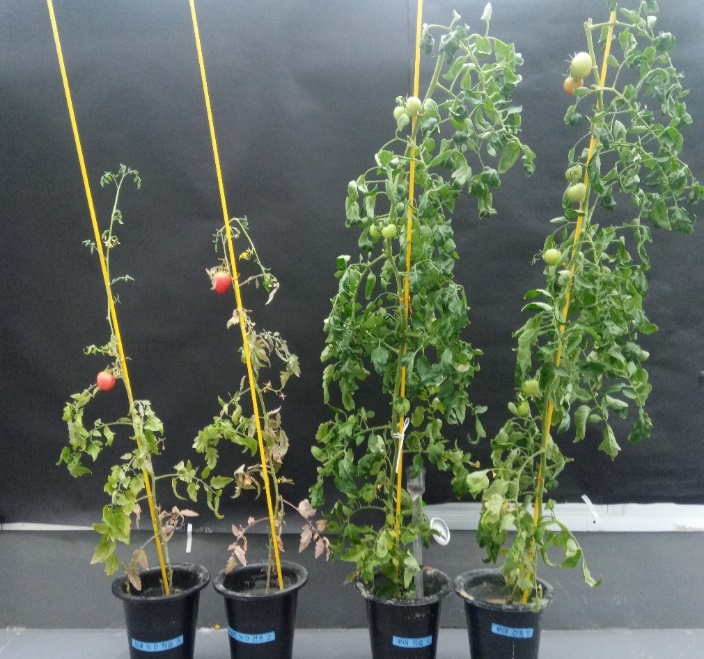  **D**  **C**  **B**  **A** | 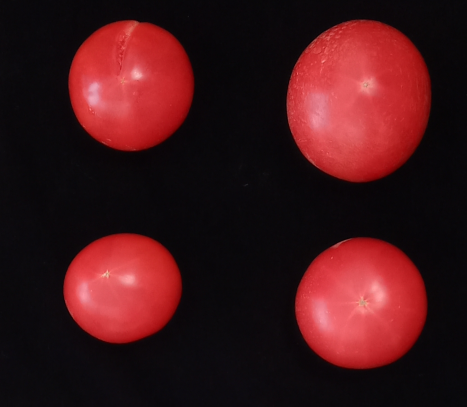  **D**  **B**  **A** |
| --- | --- |

**Figure S2.** Pictures of tomato plants and harvested fruits grown in different nutrient and water conditions (NL-WS (A); NL-WL (B); NE-WS (C); NE-WL (D)).

**C**
